# Considerations for management of interstitial ectopic pregnancies: two case reports

**DOI:** 10.1186/s13256-016-0892-9

**Published:** 2016-04-27

**Authors:** Natalia M. Grindler, June Ng, Kristina Tocce, Ruben Alvero

**Affiliations:** 1 Division of Reproductive Endocrinology and Infertility, Department of Obstetrics and Gynecology, University of Colorado Denver School of Medicine, Aurora, CO USA; 2 University of Colorado Denver School of Medicine, Aurora, CO USA; 3 Division of Family Planning, Department of Obstetrics and Gynecology, University of Colorado Denver School of Medicine, Aurora, CO USA; 4 Division of Reproductive Endocrinology & Infertility, Department of Obstetrics and Gynecology, Warren P. Alpert School of Medicine, Brown University, Providence, RI USA

**Keywords:** Cornual pregnancy, Interstitial pregnancy, Ectopic pregnancy, Persistent products of conception, Minimally invasive surgery

## Abstract

**Background:**

Conventional treatment of interstitial pregnancies includes systemic methotrexate, direct methotrexate injection, wedge resection, or hysterectomy. We present two cases of interstitial pregnancies that were successfully managed by different minimally invasive surgical techniques. We also report the novel use of hysteroscopic urologic stone retrieval forceps in the transvaginal removal of persistent products of conception after systemic methotrexate for an interstitial pregnancy.

**Case presentation:**

Case 1 was a 28-year-old gravida 1 white woman at 8 weeks gestation; she was diagnosed with a left interstitial pregnancy. After laparoscopic confirmation of the interstitial pregnancy, successful ultrasound-guided suction dilation and curettage was performed. Case 2 was a 33-year-old gravida 3 para 1021 (one term pregnancy, no preterm pregnancies, one ectopic pregnancy and one spontaneous miscarriage, and one living child) Hispanic woman with persistent products of conception after systemic methotrexate for a left interstitial pregnancy. She underwent hysteroscopic-guided removal of the persistent products of conception, which was possible due to novel use of urologic stone retrieval forceps.

**Conclusions:**

Successful minimally invasive treatment of interstitial pregnancies may be possible in certain cases. Collaboration between different specialties continues to be important for improving minimally invasive options.

## Background

A cornual ectopic pregnancy is defined as a gestation that occurs within the endometrium of the horn of a unicornuate or bicornuate uterus, whereas an interstitial pregnancy occurs within the uterus at the junction of the uterus and the proximal part of the fallopian tube [1–3]. Interstitial pregnancy occurs when implantation occurs in the most proximal section of the tube surrounded by the myometrium. This interstitial portion of the fallopian tube is highly vascular; rupture results in excessive intraperitoneal hemorrhage. Although these pregnancies represent approximately 2 to 4 % of all ectopic pregnancies, maternal mortality is high (2 % of cases) due to the risk of uterine rupture and subsequent hemorrhagic shock [1–4]. The routine use of early ultrasonography has enabled the development of minimally invasive surgical techniques before serious complications occur. The diagnosis of an interstitial pregnancy can be made based on the following ultrasound criteria: empty uterine cavity, a chorionic sac separate and at least 1 cm from the lateral edge of the uterine cavity, and a thin (<5 mm) myometrial layer surrounding the gestational sac [1, 5]. Risk factors for an interstitial pregnancy are similar to those for any ectopic pregnancy: pelvic inflammatory disease, previous tubal surgery, previous ectopic pregnancy, assisted reproductive technology, and congenital uterine anomalies. Ipsilateral salpingectomy is the only risk factor that exists exclusively for interstitial pregnancy [2].

Conventional treatment for interstitial pregnancies includes systemic methotrexate, cornual wedge resection, or hysterectomy. With the advancement of ultrasound and minimally invasive techniques, other management options now include direct injection of methotrexate into the abnormal pregnancy, combined systemic and direct injection technique [6], and laparoscopic cornual wedge resection. To the best of our knowledge, there have been no cases reported of tubal rupture from persistent products of conception (POC) in an interstitial pregnancy after surgical treatment or methotrexate. However, given the potential risk of uterine rupture with associated high mortality and morbidity, persistent POC pose a potential threat to a woman’s future fertility and overall health. We present two cases of interstitial pregnancies that were successful managed by different minimally invasive surgical techniques.

## Case presentation

### Case 1

A 28-year-old gravida 1 para 0 white woman with a past medical history significant for polycystic ovarian syndrome and a history of laparoscopic gastric bypass surgery, presented to our emergency department with vaginal bleeding. She reported clomiphene citrate use and was 8 weeks 6 days pregnant by last menstrual period. She had a benign pelvic examination. Her beta hCG level was 39,745 mIU/mL. A transvaginal ultrasound revealed an eccentrically located gestational sac with 3 mm of myometrium in the left posterior cornu. A yolk sac and embryo with a crown-rump length (CRL) of 6.2 mm were also seen but no fetal cardiac activity was demonstrated. The diagnoses of probable early pregnancy loss (EPL) and interstitial location of the pregnancy were explained to the patient. As this was a highly desired pregnancy, she declined intervention and elected for expectant out-patient management. Subsequent follow-up ultrasound 48 hours later demonstrated a CRL of 7 mm without cardiac activity and 3 mm of myometrium in the thickness area, posterior to the sac. The diagnosis of EPL of an interstitial pregnancy based on CRL >7 mm without fetal cardiac activity was discussed with her. She was counseled on all available management options including expectant and medical management. She elected to proceed with surgical management and gave consent for the following potential procedures: examination under anesthesia, dilation and curettage (D&C), diagnostic laparoscopy, possible operative laparoscopy, possible exploratory laparotomy, possible cornual wedge resection with salpingectomy, and possible hysterectomy. She was informed that D&C is not the standard treatment modality for interstitial pregnancies. However, after a family planning specialist and a radiologist reviewed her images, it was felt that the inferior aspect of the sac might be accessible with a cannula angled towards the cornua. Since this was an EPL, pre-procedure methotrexate was not offered because there was no evidence indicating that it would facilitate removal.

**Fig. 1:**
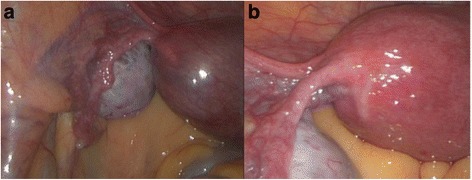
Laparoscopic intraoperative images of Case 1. **a** The left cornu of the uterus is thin and tensely distended with a gestational sac. **b** The left cornu after manual vacuum aspiration of abnormal pregnancy

Laparoscopic confirmation of her interstitial pregnancy was performed first to ensure that no interval interstitial rupture had occurred. Laparoscopy revealed the left cornu of her uterus to be thin and tensely distended with a gestational sac; a large blood vessel was seen overlying the cornu (Fig. 1). Her cervix was then dilated to 7 mm and a 7 mm flexible cannula was placed just inferior to the gestational sac under direct ultrasound guidance. Manual vacuum aspiration was performed and the POC were removed with two passes. Afterwards, her uterus appeared completely normal on laparoscopic visualization (Fig. 1). She tolerated the procedure well and was discharged home on the same day without further complications. Pathology was consistent with POC. Postoperative beta hCG were monitored weekly until <5 mIU/mL.

### Case 2

**Fig. 2:**
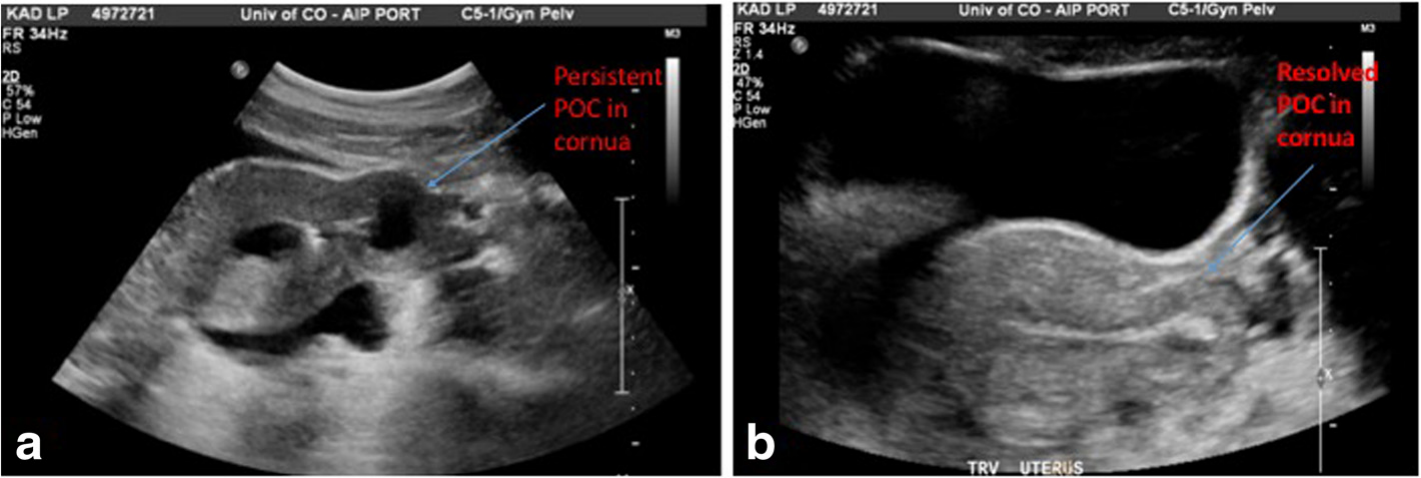
Intraoperative transabdominal ultrasound of Case 2. **a** Interstitial ectopic pregnancy with a gestational sac in the left cornu and generalized reactive muscular echogenicity surrounding the sac. **b**) After successful hysteroscopic removal, the decidualized reaction in the left cornu resolves. *POC* products of conception

A 33-year-old gravida 3 para 1021 (one term pregnancy, no preterm pregnancies, one ectopic pregnancy and one spontaneous miscarriage, and one living child) Hispanic woman with a history of a previously diagnosed cornual ectopic pregnancy in a unicornuate uterus presented for evaluation and treatment of suspected persistent POC in the left cornu of a unicornuate uterus. Her pregnancy history is notable for a term uncomplicated vaginal delivery and prior early first trimester miscarriage managed with expectant management. She had no other significant medical or surgical history. She was diagnosed with a left interstitial pregnancy and treated with systemic methotrexate, receiving a total of four doses. Given her desire to conceive again, she underwent a gynecologic ultrasound, which revealed a persistent gestational sac and fetal pole in the left cornu despite multiple confirmatory hCG values <5. She was asymptomatic but was referred to the reproductive endocrinology service for management of this residual mass given her desire to try to conceive again and the potential to utilize assisted reproductive technology in future cycles. Ultrasound revealed a gestational sac in the left cornu measuring 10×7 mm with generalized reactive muscular echogenicity surrounding the sac and a fetal pole measuring 6.2 mm (Fig. 2). Ultrasound findings were suggestive that these persistent POC would be accessible via suction D&C based on its continuity with the endometrial stripe on ultrasound.

She was counseled regarding the following options: further expectant management with serial ultrasound monitoring given no known evidence that persistent POC can result in uterine rupture, and surgery. She elected to proceed with definitive surgical treatment given her desire to conceive and the potential catastrophic nature of uterine rupture in her unicornuate uterus. Intraoperative transvaginal and transabdominal ultrasound again confirmed persistent left interstitial pregnancy with POC within surrounding decidualized endometrium. A family planning specialist was consulted during this case. First, suction D&C was attempted. A 7 mm flexible cannula was inserted to the fundus under transvaginal ultrasound guidance. The left cornu was unable to be accessed despite multiple attempts with transabdominal and transvaginal ultrasound guidance. This was also attempted with a 7 mm rigid curved cannula but similarly was unsuccessful.

**Fig. 3:**
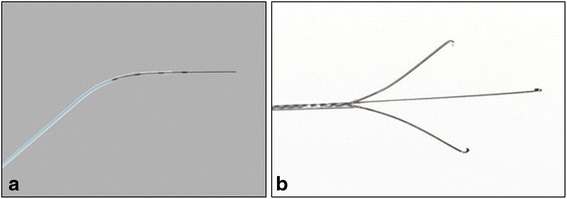
**a** Novy™ Cornual Cannulation Set (Cook Medical) with 5.0-Fr sheath and 0.46 mm guide wire diameter and **b** Tricep™ extra-strength hooked-prong grasping forceps (Boston Scientific) with a 3.0-Fr sheath and 120-cm working length (urologic stone retrieval basket) utilized for management in Case 2

**Fig. 4:**
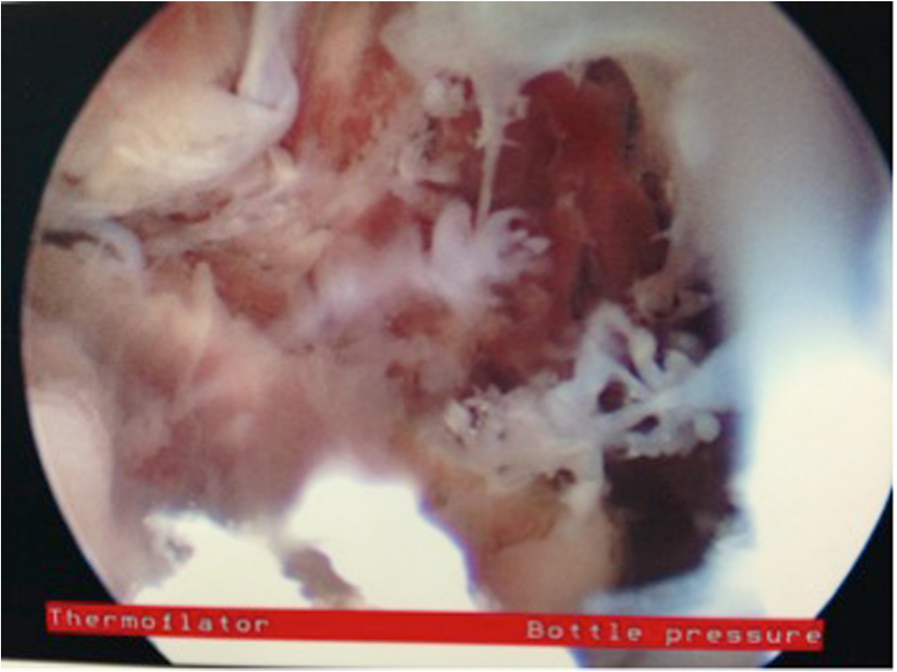
Hysteroscopic visualization of persistent products of conception from within the cornu after treatment with systemic methotrexate for cornual ectopic pregnancy in Case 2

**Fig. 5:**
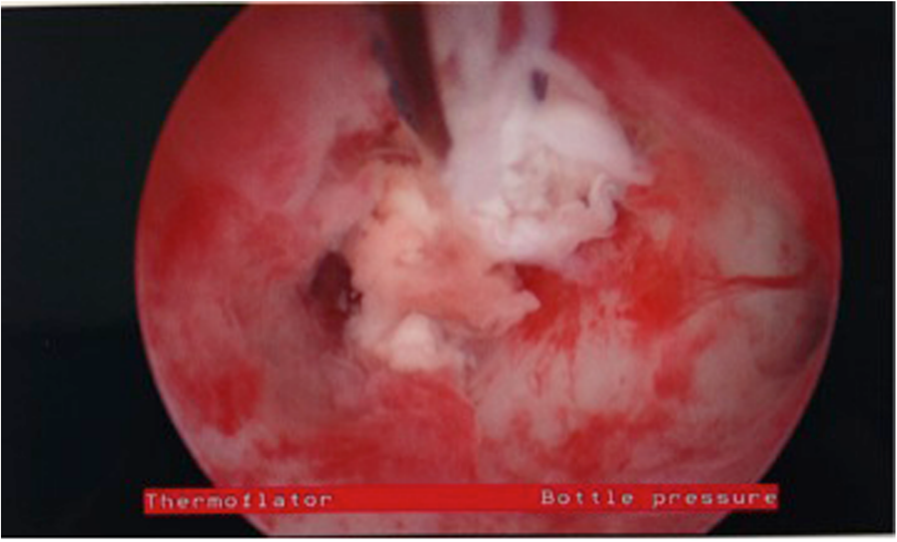
Persistent products of conception being removed from the cornu with the use of urologic stone retrieval forceps in Case 2

Hysteroscopy was then performed using a 5 mm Karl Storz operative hysteroscope with a 2.9 mm 30° lens, 5mm sheath, and 5-Fr operating port with normal saline as the distending media. Upon entry of the hysteroscope into her uterus, tubal ostium was identified. However, no gross POC were visualized within her uterine cavity. Next, a Novy™ Cornual Cannulation Set (Cook Medical) was passed hysteroscopically into the left cornual region under hysteroscopic and ultrasound guidance (Fig. 3). Although the device was successfully cannulated into the left cornu, no tissue was able to be aspirated (Fig. 4). As an alternative intended to grasp and remove the POC, a Tricep™ extra-strength hooked-prong grasping forceps (Boston Scientific) with a 3.0-Fr sheath and 120-cm working length (urologic stone retrieval basket) was suggested based on prior cornual procedures with a similar device [7]. This device was placed into the left cornu under both hysteroscopic and ultrasound guidance (Fig. 3); it was opened and closed within the cornual region several times. Dense fibrous tissue was grasped and removed with visible POC. This was repeated several times in a similar fashion. Karl Storz 5-Fr hysteroscopic grasping forceps were also used to grasp tissue extruding from this cornu several times (Fig. 5). At the end of the case, ultrasound revealed resolution of the myometrial decidual reaction with removal of persistent POC (Fig. 2). Safety was assured during the case with constant hysteroscopic visualization; transabdominal ultrasonography was also utilized to assure excellent visualization at all times. Laparoscopy was not necessary in this case due to combined hysteroscopic and ultrasonographic visualization during the case. The patient was counseled that the resolution of the persistent POC on ultrasound and hysteroscopy decreased the risk of uterine rupture but she was cautioned regarding the continued potential for rupture. Pathology revealed fragments of necrotic chorionic villi and decidua. She was discharged home the same day and had an uncomplicated postoperative course. Postoperative beta hCG was measured again postoperatively and was <5 mIU/mL.

## Discussion

Cornual and interstitial pregnancies are rare types of ectopic pregnancies, but remain a significant cause of maternal morbidity and mortality. In our first case, collaboration between a radiologist and a family planning specialist resulted in the successful resolution of an interstitial pregnancy with the use of ultrasound-guided suction D&C. A flexible cannula was chosen in order to minimize the risk of cornual perforation. With this instrument, the cornu did not need to be directly instrumented (the cannula was placed just inferior to the sac) and the manual vacuum aspiration suction minimized potential trauma to the cornu because as the device fills with tissue, the force of the suction decreases.

Our second case resulted in collaboration between family planning and reproductive endocrinology specialists and led to the successful resolution of persistent cornual POC. Hysteroscopic-guided curettage using urologic instruments was utilized in this case. The presence of the cornual abnormality, which could be detected both by ultrasound and hysteroscopically in our second case, dictated the need to remove the tissue for histologic evaluation. Her undetectable hCG was probably due to the necrotic nature of the chorionic villi which was confirmed histologically. The cornu was normalized by the conclusion of the case and the patient currently has an ongoing normal pregnancy. In both cases, interstitial pregnancies were successfully managed with minimally invasive techniques that were fertility-preserving surgical techniques, which resulted in minimal blood loss, preserved reproductive organs, and a probable return to normal reproductive function. Advanced minimally invasive techniques and innovative approaches to management of the unruptured interstitial pregnancy in our patients resulted in optimal immediate postoperative outcomes.

There is no general consensus on the best surgical procedure for interstitial ectopic pregnancy. Medical treatment with methotrexate has been associated with a failure rate ranging from 9 to 65 % [3, 8–10] and the best medical treatment regimen for interstitial pregnancy remains unknown. Increasingly more conservative approaches are being used due to the proliferation of minimally invasive gynecological surgery and advanced surgical techniques. Our criteria for offering minimally invasive surgical techniques include the following: hemodynamic stability and no evidence of uterine rupture. Although successful hysteroscopic management of interstitial pregnancies has previously been reported [11], we believe that our novel utilization of urologic forceps has the potential to be applied to similar and related cases. Use of a 8F pediatric catheter has been reported for transcervical suction of POC in interstitial pregnancies while under laparoscopic visualization; however, at least two cases utilizing this technique have resulted in cornual perforation [12]. Similarly, use of hysteroscopic polyp forceps under ultrasound guidance resulted in incomplete extraction and postoperative methotrexate was required [13]. Other minimally invasive techniques described for management of interstitial pregnancies include laparoscopic-guided transcervical evacuation [14], laparoscopic-guided use of a resectoscope [15], ipsilateral uterine artery ligation at the time of cornual repair [16, 17], and the use of end loop and encircling sutures at the cornua [16, 18–20]. Our cases highlight the importance of collaboration between different medical specialties in these challenging rare clinical cases as well as the importance of attempting novel techniques in appropriately selected patients when the alternative would result in unnecessary additional risk for the patient.

As is the case for all novel minimally invasive techniques, the surgeon should be ready to perform cornual resection with salpingectomy or hysterectomy if uterine perforation occurs. The risk of uterine rupture during subsequent pregnancies in patients who have been previously treated for an interstitial pregnancy has not been clearly established [3]. Although the exact risk of uterine rupture in subsequent pregnancy is unknown, women should be counseled carefully about this possibility as uterine rupture has occurred in patients with previous treatments for interstitial pregnancies [21, 22]. These cases highlight that interstitial pregnancies can be managed with minimally invasive procedures in properly selected patients.

## Conclusions

In this case report, we present two cases of interstitial pregnancies that were successfully managed with minimally invasive techniques, including the novel use of hysteroscopic urologic stone retrieval forceps to remove retained cornual tissue. The approaches utilized in these cases were the result of collaboration between subspecialties, with the goal of preserving fertility that was strongly desired by these patients. Subsequent pregnancies should be closely monitored because of the risks of recurrent ectopic pregnancy and theoretical uterine rupture. Whenever possible, non-invasive management of interstitial pregnancies should be attempted. Success leads to better immediate and possible future outcomes that are important for the reproductive health of our patients.

## Consent

Written informed consent was obtained from the patients for publication of this case report and accompanying images. A copy of the written consents is available for review by the Editor-in-Chief of this journal.

## Abbreviations

CRL: crown-rump length
D&C: dilation and curettage
EPL: early pregnancy loss
POC: products of conception

## References

[1] Moawad NS, Mahajan ST, Moniz MH, Taylor SE, Hurd WW. Current diagnosis and treatment of interstitial pregnancy. Am J Obstet Gynecol. 2010;202(1):15–29. 10.1016/j.ajog.2009.07.054 20096253

[2] Tulandi T, Monton L. Conservative surgical management of interstitial pregnancy. Fertil Steril. 1990;53(3):581. 10.1016/s0015-0282(16)53364-0 2407570

[3] Tulandi T, Al-Jaroudi D. Interstitial pregnancy: results generated from the Society of Reproductive Surgeons Registry. Obstet Gynecol. 2004;103(1):47–50. 10.1097/01.AOG.0000109218.24211.79 14704243

[4] Fabre-Gray A, Read M, Wardle P, James M. Recurrent cornual pregnancy, successfully treated with methotrexate, following a ruptured pregnancy in the contralateral cornu. J Obstet Gynaecol. 2014;34(1):85. 10.3109/01443615.2013.819844 24359059

[5] Timor-Tritsch IE, Monteagudo A, Matera C, Veit CR. Sonographic evolution of cornual pregnancies treated without surgery. Obstet Gynecol. 1992;79(6):1044–9. 1579304

[6] Swank ML, Harken TR, Porto M. Management of interstitial ectopic pregnancies with a combined intra-amniotic and systemic approach. Obstet Gynecol. 2013;122(2 Pt 2):461–4. 10.1097/AOG.0b013e31828d58ee 23884260

[7] Goldthwaite LM, Edwards L, Tocce K. Early hysteroscopic removal of intratubal microinserts with urologic stone retrieval forceps. Obstet Gynecol. 2014;124(2 Pt 2 Suppl 1):441–4. 10.1097/AOG.0000000000000338 25004312

[8] Buster JE, Heard MJ. Current issues in medical management of ectopic pregnancy. Curr Opin Obstet Gynecol. 2000;12(6):525–7. 10.1097/00001703-200012000-00012 11128417

[9] Hiersch L, Krissi H, Ashwal E, From A, Wiznitzer A, Peled Y. Effectiveness of medical treatment with methotrexate for interstitial pregnancy. Aust N Z J Obstet Gynaecol. 2014;54(6):576–80. 10.1111/ajo.12251 25338827

[10] Warda H, Mamik MM, Ashraf M, Abuzeid MI. Interstitial ectopic pregnancy: conservative surgical management. JSLS. 2014;18(2):197–203. 10.4293/108680813X13753907292836 PMC4035629 24960482

[11] Nezhat CH, Dun EC. Laparoscopically-assisted, hysteroscopic removal of an interstitial pregnancy with a fertility-preserving technique. J Minim Invasive Gynecol. 2014;21(6):1091–4. 10.1016/j.jmig.2014.04.007 24768982

[12] Cai Z, Wang F, Cao H, Xia Q, Chen X, Cai Y. The value of laparoscopy alone or combined with hysteroscopy in the treatment of interstitial pregnancy: analysis of 22 cases. Arch Gynecol Obstet. 2012;285(3):727–32. 10.1007/s00404-011-2060-1 21874409

[13] Ahn JW, Lee SJ, Lee SH, Kang SP, Won HS. Ultrasound-guided transcervical forceps extraction of unruptured interstitial pregnancy. BJOG. 2013;120(10):1285–8. 10.1111/1471-0528.12265 23683343

[14] Fritz RB, Rosenblum N, Gaither K, Sherman A, McCalla A. Successful laparoscopically assisted transcervical suction evacuation of interstitial pregnancy following failed methotrexate injection in a community hospital setting. Case Rep Obstetr Gynecol. 2014;2014:695293. 10.1155/2014/695293 PMC3932726 24649387

[15] Minelli L, Landi S, Trivella G, Fiaccavento A, Barbieri F. Cornual pregnancy successfully treated by suction curettage and operative hysteroscopy. BJOG. 2003;110(12):1132–4. 14664889

[16] Radwan Faraj MS. Review: management of cornual (interstitial) pregnancy. Obstetr Gynaecol. 2007;9:249–55.

[17] Khawaja N, Walsh T, Gill B. Uterine artery ligation for the management of ruptured cornual ectopic pregnancy. Eur J Obstet Gynecol Reprod Biol. 2005;118(2):269. 10.1016/j.ejogrb.2004.05.006 15653221

[18] Tulandi T, Vilos G, Gomel V. Laparoscopic treatment of interstitial pregnancy. Obstet Gynecol. 1995;85(3):465–7. 10.1016/0029-7844(94)00423-B 7862394

[19] Moon HS, Choi YJ, Park YH, Kim SG. New simple endoscopic operations for interstitial pregnancies. Am J Obstet Gynecol. 2000;182(1 Pt 1):114–21. 10.1016/s0002-9378(00)70499-6 10649165

[20] Shendy M, Atalla R. Modern management of cornual ectopic pregnancy. In: Kamrava M, editor. Ectopic pregnancy – modern diagnosis and management. Croatia: InTech; 2011. p. 238–48.

[21] Budnick SG, Jacobs SL, Nulsen JC, Metzger DA. Conservative management of interstitial pregnancy. Obstet Gynecol Surv. 1993;48(10):694–8. 10.1097/00006254-199310000-00025 8247463

[22] Weissman A, Fishman A. Uterine rupture following conservative surgery for interstitial pregnancy. Eur J Obstet Gynecol Reprod Biol. 1992;44(3):237–9. 10.1016/0028-2243(92)90105-8 1607064

